# Factors Contributing to Noncompliance With Diabetic Medications and Lifestyle Modifications in Patients With Type 2 Diabetes Mellitus in the Eastern Province of Saudi Arabia: A Cross-Sectional Study

**DOI:** 10.7759/cureus.31965

**Published:** 2022-11-28

**Authors:** Mohammed R Alfulayw, Raghad A Almansour, Sarah K Aljamri, Asia H Ghawas, Sarah S Alhussain, Abdulaziz A Althumairi, Ahmed A Almuthaffar, Khalid A Alhuwayji, Atheer A Almajed, Samia S Al-Yateem, Abdullah S Alamri, Noura H Alhussaini, Malak A Almutairi, Abdulrahman O Alali, Abdulrahman F Alkhateeb

**Affiliations:** 1 Medical School, Imam Abdulrahman Bin Faisal University, Dammam, SAU; 2 Medical School, Imam Mohammed ibn Saud University, Riyadh, SAU; 3 General Practice, Dhurma General Hospital Riyadh Cluster 3, Riyadh, SAU; 4 Medical School, Arabian Gulf University College of Medicine, Manama, BHR; 5 Medical School, King Faisal University, Al-Ahsa, SAU; 6 Medical School, Ibn Sina National College, Jeddah, SAU; 7 Medical School, Johns Hopkins Aramco Healthcare, Dhahran, SAU; 8 General Practice, King Abdulaziz Hospital, Ministry of National Guard Health Affairs (MNGHA), Al-Ahsa, SAU; 9 Family Medicine, Ministry of Health, Ibn Hayyan Pharmaceuticals, Dammam, SAU

**Keywords:** physical activity, lifestyle, adherence, antidiabetic therapy, diabetes mellitus type 2

## Abstract

Background: Type 2 diabetes mellitus (T2DM) is defined as a chronic medical condition in which the blood glucose level remains high. The risk factors of T2DM are high body mass index due to obesity or being overweight, genetics, and certain medical conditions. Lifestyle modification plays a crucial role in T2DM regulation and prevention, and if it is not controlled well by either lifestyle modification or DM regulatory medications, it may lead to medical complications ranging from mild to life-threatening complications.

Aim: The purpose of this study is to find the contributory factors of noncompliance with oral antidiabetic drugs and lifestyle modifications in patients with T2DM in the eastern province of Saudi Arabia. This will help control one of the most widespread comorbidities that might otherwise be a significant burden on patients’ health and financial status as well as on the government.

Methodology: A cross-sectional questionnaire study was conducted on T2DM patients in the eastern province of Saudi Arabia through a link distributed on social media, and the contributory factors of noncompliance to diabetes medication and lifestyle modification were evaluated.

Results: A total of 426 participants were included in the study. Regarding compliance with DM medications, 199 (46.7%) participants were adherent to their medications, 148 (34.7%) were not adherent to their medication, 42 (9.9%) were sometimes adherent, and 37 (8.7%) were mostly adherent to their medication. Regarding lifestyle modification, the level of adherence to a healthy diet and exercise among T2DM patients in the eastern province was low and unsatisfactory. According to the participants, the most reported factors contributing to noncompliance with DM medications and lifestyle modifications were forgetfulness, lack of knowledge about diabetes and the importance of controlling it, side effects of the medications, and difficulty in following a healthy diet. Regarding the influence of sociodemographic variables on the level of adherence in T2DM patients, factors such as age, marital status, occupation, comorbidities, diagnosis period, and previous complaints of DM complications showed significant associations with compliance with DM medication.

Conclusion: The findings of this study revealed that the level of adherence to DM medications among T2DM patients in the eastern province was suboptimal. Although free medicines were available with a high level of healthcare access through government primary healthcare centers (PHCCs), poor adherence was observed. This study highlighted that medication adherence might be affected by age, marital status, occupation, chronic diseases, diagnosis period, and previous complaints of DM complications. Regarding lifestyle modification, this study showed that the level of adherence to a healthy diet and exercise among T2DM patients in the eastern province was low and unsatisfactory. Our recommendation is to measure the presence of dietician clinics, patient relationships with their healthcare providers, and their effect on patient compliance with DM medications. Further research is needed to include other factors that could influence adherence, such as patient-healthcare provider communication. Moreover, it is suggested that PHCCs discuss with noncompliant patients the reasons that prevent them from adhering to their medication and lifestyle modifications as part of their care plan.

## Introduction

Type 2 diabetes mellitus (T2DM) is defined as a chronic metabolic disorder characterized by persistent high blood sugar levels [1]. The pathophysiology of T2DM is due to resistance to peripheral actions of insulin, impaired insulin secretion, or both [1]. T2DM is a serious condition that can cause many complications if there is poor glycemic control because of noncompliance with DM medications or lifestyle medications [1]. It can cause damage to various organ systems, leading to the development of life-threatening complications, most commonly microvascular and macrovascular complications, which increase the risk of cardiovascular diseases by approximately two- to fourfold [1].

T2DM is a growing global health concern [2]. The estimated global diabetes prevalence in the adult population was 9.3% (463 million) in 2019, and it is projected to increase to 10.9% (700 million) by 2045 [2]. Half of the 463 million people living with diabetes were undiagnosed with diabetes or unaware of their status [2]. In 2021, among adults, one out of every 10 people was living with diabetes [3].

The costs of diabetes pose a huge financial burden on people living with diabetes and even on the government [3]. The health expenditures were 232 billion USD in 2007, and this number increased to 966 billion USD in 2021 for adults aged from 20 to 79 years, which is a 316% increase over 15 years, and the costs are expected to increase in the next years [3].

Prediabetes or impaired glucose tolerance is a health condition in which blood glucose levels are above the normal range and below the T2DM stage [3]. In 2021, 10.6% of adults worldwide (541 million) were expected to have prediabetes [3].

One of the risk factors of T2DM is gestational diabetes mellitus (GDM), which has recently become an increasing health concern due to its effect on the mother and the child. A study found that the prevalence of GDM increased from 4.6% in 2006 to 8.2% in 2016 among women in the United States [4].

Furthermore, according to the World Health Organization (WHO), it has been estimated that 7 million Saudis have diabetes and at least 3 million have prediabetes, ranking Saudi Arabia as having the second-highest rate of diabetes in the Middle East (seventh highest worldwide) [5]. Saudi Arabia is expected to have 7.5 million cases of T2DM by 2035 [5], which is a twofold increase from the 2015 levels. In addition, there has been an increase in healthcare expenses and diabetes treatment in Saudi Arabia by more than 500% over the past two decades [5]]. Therefore Saudi Arabia faces serious public health concerns, including a rising prevalence of diabetes, micro- and macrovascular complications, and delayed diagnosis [5].

As a result, the DM burden in Saudi Arabia will likely grow to very serious levels unless the nation enacts a comprehensive epidemic control program and adopts a multidisciplinary approach [6]. People with diabetes must be treated in such a way that they achieve improved health and a higher quality of life, thereby decreasing the personal and social expenses for diabetes care [6]. Several studies have found that diabetes education programs have improved glycemic control in patients with T2DM [6]. Moreover, engaging in multifactorial diabetes education programs has shown significant improvements in patients’ glycemic and lipid levels in the short term, particularly among those with adverse glycated hemoglobin (HbA1c) or low-density lipoprotein (LDL) levels before participating [6].

Strategies for managing diabetes often rely on lifestyle modifications, including dietary interventions, exercise, and pharmacological approaches [7]. Many studies have reported that intensive nutritional intervention by itself or in addition to medications is the best method for improving glycemic control in patients with T2DM [7]. There is also evidence that consuming diets with a high glycemic index and glycemic load over a long period may have implications for metabolism and health, including chronic hyperglycemia and hyperinsulinemia, which can lead to insulin resistance and diabetes [7].

A study was conducted on individuals newly diagnosed with T2DM, which combined qualitative methods with self-determination theory to identify the motivational experiences of people newly diagnosed with T2DM [7]. Initial behavioral changes were reported by participants with relatively dominant controlled motivation, but this was often accompanied by internal conflict, frustration, and a constant need for external prompting [7]. Moreover, those with autonomous motivation approached behavior change more flexibly and incorporated it into a new way of life [7]. They also desired continual support for self-regulation [7]. Considering these findings, it is important to understand the quality of motivation in this group and carefully consider the types of motivation targeted in lifestyle interventions for people with T2DM [7].

A cross-sectional study was conducted in Hodeida City, Yemen. A total of 210 participants were included in the study, and 54.8% of them were males [8]. The study showed that the rates of adherence to diet and exercise were 21.0% and 15.2%, respectively [8]. Furthermore, it revealed that only 21.0% of patients adhered to the prescribed diet and 15.0% adhered to regular exercise [8]. However, physical exercise adherence rates varied between 9.5% and 35.6% [8]. The study concluded that adherence to diet and physical exercise among T2DM patients in Yemen was still low. The low adherence rate was measured through the level of glycated hemoglobin (HbA1c) [8].

A study conducted in 2019 to assess adherence and glycemic control in Saudi Arabia showed that 42.8% of the participants were not in compliance with DM medications [9]. Another study conducted in 2019 in al Khobar city to assess adherence to DM medications reported that only a few patients (35.8%) had high adherence to antidiabetic medications [10].

A study conducted in the Al Hasa region in the eastern province of Saudi Arabia showed that the rate of noncompliance among diabetic patients was high (65%-69%) [11]. It showed that the main causes for nonadherence were the appointment schedule, physical activity, and diet [11]. Also, the unavailability of transport and forgetfulness were the main reasons that patients could not see the doctor on the day of their appointment [11].

Research conducted in the Bisha region in Saudi Arabia aimed to assess medication adherence among patients with diabetes and associated factors in Bisha primary healthcare centers (PHCCs) in Saudi Arabia [12]. The level of adherence to medication in patients with DM in the Bisha PHCCs was found to be suboptimal [12]. Adherence to diabetic medications was found to be positively associated with a decrease in HgA1c levels [12].

In the Jazan region in Saudi Arabia, the rate of adherence and the factors contributing to compliance among diabetic patients were measured [13]. The study showed that only 23% of participants reported good medication adherence [13]. the following factors were found to be significantly associated with adherence: a place of residency, distance from the healthcare center, patients who regularly attend appointments, and HbA1c > 8 [13].

Another study aimed to assess adherence to oral hypoglycemic medication among patients with diabetes in Saudi Arabia reported that 54.8% of participants had a low adherence level [14]. The three main factors that may contribute to nonadherence to medication were nonadherence to regular follow-ups in the diabetes clinic, nonadherence to a healthy diet, and nonadherence to instructions to take medication [14].

DM is a serious widespread condition that is increasing at an alarming rate in Saudi Arabia. On a personal level, we noticed that a vast percentage of diabetic patients attending PHCCs had an uncontrolled level of blood glucose. This prompted us to think about the factors or reasons that could influence and be decisive in the patient’s journey with T2DM. Investigating the factors that contribute to noncompliance with DM medications and lifestyle modifications will help control one of the most costly and common chronic diseases in Saudi Arabia.

## Materials and methods

Study design

An observational cross-sectional questionnaire study was conducted to evaluate the factors that contribute to noncompliance with diabetes medication and lifestyle modification in T2DM patients in the eastern province of Saudi Arabia.

Study subjects

The study subjects were T2DM patients on oral antidiabetic medications, and they consented to participate in the study for free, without any monetary rewards or incentives. They filled out the questionnaire during the study period while meeting the inclusion and exclusion criteria.

Criteria for patient selection

The inclusion and exclusion criteria used in this study are illustrated in Table 1.

**Table 1:** Inclusion and exclusion criteria.

Criterion	Inclusion	Exclusion
Age	Patients aged 18 years or older	Patients aged less than 18 years
Diseases	Type 2 diabetic patients with hypertension, ischemic heart disease, and dyslipidemias	Patients with gestational diabetes or mental incompetents
Medication	Patients who are on oral antidiabetic medications plus insulin	Patients who are not using oral medications

Sample size and sampling

Convenient random sampling was used for data collection. The minimally required sample size was calculated using the formula n = z2pq\d2 [15], with a confidence level of 95%, an estimated proportion of 50%, a 5% level of precision, and an estimated population of 741,398 (according to WHO, the prevalence of diabetes in Saudi Arabia is 14.4%, and the population of the eastern region of Saudi Arabia is 5,148,598 according to the authority of statistics in Saudi Arabia, which makes the estimated number of diabetic patients in the eastern region of Saudi Arabia 741,398) [16,17]. The minimum sample size was calculated to be 384; however, a higher number was included in the study to ensure accuracy.

Study tool and its validation

To use a validated study tool, a multiple-step process was followed. First, the study tool (the survey) was constructed by the investigators under the supervision of the primary investigator (a consultant in family medicine) and after reviewing several study surveys that were similar to ours. Then, the questionnaire was presented to multiple consultants experienced in the field to revise it and ensure that it covered the goals of the study and that its content was appropriate. The survey was revised and modified, and then its use was affirmed by the consultants. The questionnaire was first established in English and then presented to a language expert to translate into Arabic. After the translation, it was given to another language expert to translate it back to Arabic to ensure the precision of the translation. The survey was linguistically approved after minor grammatical and linguistic editing in both Arabic and English. Lastly, a pilot study was launched on a small group of people (18 persons) to ensure that there was a uniform understanding of the survey content.

Data collection methods

Data were collected by offering an online link to the questionnaire where the patients could self-fill the questionnaire and distribute it through social media.

This questionnaire was composed of different sections that assessed various domains:

1. Sociodemographic data: age, gender, marital status, educational level, place of residency, occupation, monthly income, type of healthcare received, and type of health insurance, if present

2. Medical history: onset of the disease, body mass index (BMI), complications, chronic comorbidities, exercise history, and diet

3. Participants’ current compliance with antidiabetic medication taking

4. Status of diabetes control at the time of data collection (HbA1c)

5. Factors they thought to contribute to noncompliance with diabetes medication.

Data management and statistical analysis

Data were collected initially in Google forms; then, they were coded into a Microsoft Excel sheet. Next, data were transferred for analysis in the Statistical Package for the Social Sciences (SPSS), 23rd version. Frequency and percentages were used to display categorical variables. The mean and standard deviation were used to present continuous variables. Independent t-test, ANOVA test, and chi-square test were employed to test for the association as appropriately. The level of significance was set at 0.05.

Confidentiality and ethical consideration

All participants consented to participate in the study. Data were managed with confidentiality, and privacy was ensured throughout all steps of the study. Ethical approval was obtained from the Saudi Ministry of Health’s Ethical Board (No. H-02-J-002).

## Results

A total of 426 participants were included in the study. Table 2 shows the sociodemographics of the participants. As for age, 88 (20.7%) of the participants were 18-30 years, 53 (12.4%) were 31-40 years, 84 (19.7%) were 41-50 years, and 201 (47.2%) were 51 years and older. Regarding gender, 200 (46.9%) were males, while 226 (53.1%) were females. As for marital status, 79 (18.5%) were single, 319 (74.9%) were married, and 21 (4.9%) were widowed. As for education, 21 (4.9%) had an elementary school education, 40 (9.4%) had an intermediate school education, 104 (24.4%) had a high school education, 240 (56.3%) had a college education, and 21 (4.9%) had a post-graduate education. As for occupation, 44 (10.3%) were students, 127 (29.8%) were employees, 111 (26.1%) were unemployed, and 144 (33.8%) were retired. As for monthly income, 108 (25.4%) had an income of less than 4,000 SR, 99 (23.2%) had an income between 4,000 and 8,000 SR, and 219 (51.4%) had an income of more than 8,000 SR. As for the place of residency, the places with the highest rate of participants were Al Asha (163, 38.3%), Ad Dammam (104, 24.4%), and Qatif (48, 11.3%).

**Table 2:** Sociodemographic characteristics of the participating diabetics (n = 426). ^1^: Include single (79; 18.5%), divorced (7; 1.6%), and widow (21; 4.9%).

Variables		n=426	%
Age (years)	18 - 30 years	88	20.70
	31 - 40 years	53	12.40
	41 - 50 years	84	19.70
	51 years and older	201	47.20
Gender	Male	200	46.90
	Female	226	53.10
Marital status	Married	319	74.90
	Unmarried^1^	107	25.1
Place of Residency	Al Ahsa	163	38.30
	Ad Dammam	104	24.40
	Qatif	48	11.30
	Al Khobar	46	10.80
	Al Dhahran	27	6.30
	Jubail	24	5.60
	Hafer al batin	12	2.80
	Ras Tanura	2	0.50
Educational level	Elementary school	21	4.90
	Intermediate school	40	9.40
	High school	104	24.40
	College	240	56.30
	Post-graduate	21	4.90
Occupation	Student	44	10.30
	Employee	127	29.80
	Unemployed	111	26.10
	Retired	144	33.80
Monthly Income	Less than 4000 SR	108	25.40
	4000 - 8000 SR	99	23.20
	More than 8000 SR	219	51.40
Medical Insurance	No insurance	222	52.1
	Partial insurance	73	17.1
	Full insurance	131	30.8
Healthcare type	Public health care	321	75.4
	Private healthcare	105	24.6

**Figure 1:**
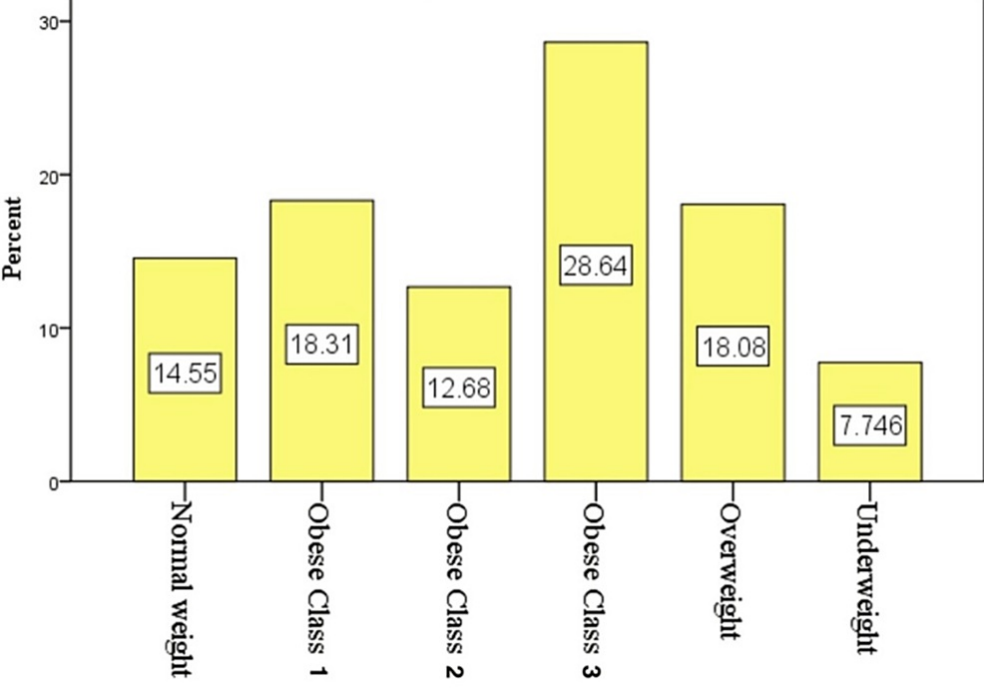
Classification of participating diabetics according to BMI.

The BMI of the participants is shown in Figure 1 as follows: 33 (7.74%) had a BMI less than 18, 62 (14.55%) had a BMI between 18 and 24, 77 (18.08%) had a BMI between 25 and 29, 78 (18.31%) had a BMI between 30 and 34, 54 (12.68%) had a BMI between 35 and 39, and 122 (28.64%) had a BMI of 40 or higher.

Table 3 illustrates the diabetic profile of the participants. As for the level of HbA1c the last time the participants measured it, 75 (17.6%) had HbA1c less than 7, 149 (35%) had HbA1c higher than 7, and 202 (47.4%) reported that they were not sure how much it was. Only 148 (34.7%) reported following a healthy diet. As for the frequency of exercise, 49 (11.5%) exercised daily, 137 (32.2%) exercised three to four days per week, and 240 (56.4%) were not exercising. Of the participants, 116 (27.2%) reported experiencing diabetes complications before, while 310 (72.8%) did not.

**Table 3:** Diabetic profile of the participants (n = 426). ^1^: A total of 109 (25.6%) had hypertension, 44 (10.3%) had high cholesterol, 22 (5.2%) had mental/psychological illness, and 15 (3.5%) had osteoporosis.

Medical history and lifestyle		No			%
Chronic diseases	Yes ^1^		190	44.6	
	No		236	55.4	
Diagnosis period	less than one year		162	38.0	
	From 1 to 5 years		104	24.4	
	From 6 to 10 years		50	11.7	
	More than 10 years		110	25.8	
Hemoglobin A1c level in the last time measured	Less than 7		75	17.6	
	7 and higher		149	35	
	Not sure		202	47.4	
Following a healthy diet	Yes		148	34.7	
	No		278	65.3	
Frequency of exercise	Daily		49	11.5	
	3 - 4 days per week		137	32.2	
	No exercise		240	56.3	
Previously complaining of diabetes complications	Yes		116	27.2	
	No		310	72.8	
Adherence to Diabetes Medications	Yes		199	46.7	
	Mostly		37	8.7	
	Sometimes		42	9.9	
	No		148	34.7	

**Figure 2:**
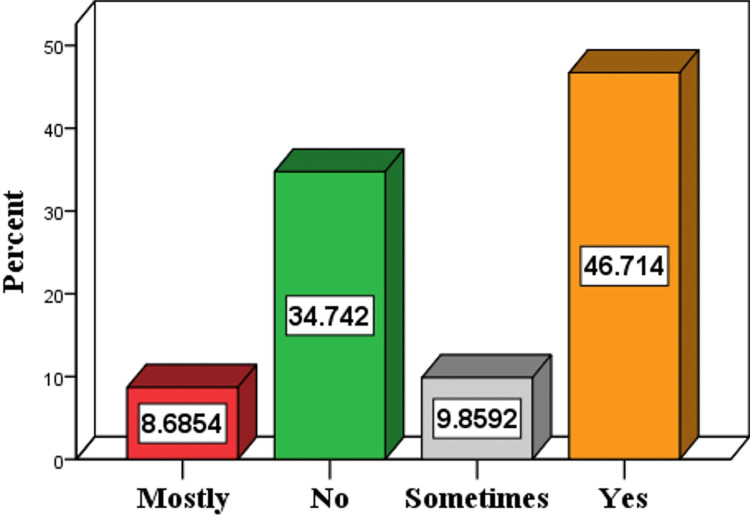
Classification of participating diabetics according to adherence to diabetes medications.

Figure 2 shows the participants’ responses toward “Adherence to Diabetes Medications,” 199 (46.7%) were adherent to their medications, 148 (34.7%) were not adherent to their medication, 42 (9.9%) were sometimes adherent, 37 (8.7%) were mostly adherent to their medication.

Table 4 displays the factors contributing to nonadherence to diabetes medications and lifestyle modification in T2DM. The most reported factors were forgetfulness (188, 44.13%), lack of knowledge about diabetes and the importance of controlling it (173, 40.61%), side effects of the medications (169, 39.67%), and hard-to-follow on a healthy diet (164, 38.5%). The least commonly reported factors were difficulty in getting the medication and the medication not being available (34, 7.98%) and prescriptions being not clear enough (36, 8.45%).

**Table 4:** Participants' opinion of the factors that contribute to non-adherence to diabetes medications and lifestyle modification in patients with type 2 diabetes.

Question	n	%
Forgetfulness	188	44.13
Lack of knowledge about diabetes and the importance of controlling it	173	40.61
Side effects of the medications	169	39.67
Hard to follow on healthy diet	164	38.50
Lack of desire and motivation to exercise	151	35.45
Lack of psychological support	137	32.16
Lack of knowledge about what is a healthy diet is	130	30.52
Medication rumors and fear	125	29.3
No time for exercise	116	27.23
Exercise intolerance	107	25.12
Altered dose of medication to suite on needs	85	19.95
Hard Follow up and no appointments available.	71	16.67
Stopped medication because didn’t think it was helping	49	11.50
Prescriptions do not clear enough	36	8.45
Hard to get the medication and it’s not available	34	7.98

Table 5 shows the influence of sociodemographic variables on the level of adherence in T2DM patients. There was a significant association between age and adherence to diabetes medications (p = 0.000), where it was observed that the older the participants, the higher the rate of adherence. There was no significant association between gender and adherence to diabetes medications. There was a significant association between marital status and adherence to diabetes medications (p = 0.000), where it was observed that widow participants (3.3%) followed by single participants (2.6%) have a higher rate of adherence. There was no significant association between the place of residency or educational level and adherence to diabetes medications. There was a significant association between occupation and adherence to diabetes medications (p = 0.000), where it was observed that retired participants (23.5%) followed by unemployed participants (12%) have a higher rate of adherence. The monthly income, medical insurance, and healthcare type were significantly associated with adherence to diabetes medications. There was a significant association between having chronic diseases and adherence to diabetes medications (p = 0.000), where it was observed that participants who have chronic diseases (27.7%) have a higher rate of adherence than those who do not have chronic diseases (19%). There was a significant association between the diagnosis period and adherence to diabetes medications (p = 0.000), where it was observed that participants who were diagnosed for more than 10 years (18.3%) had a higher rate of adherence. BMI, HbA1c, following a healthy diet, and frequency of exercise were significantly associated with adherence to diabetes medications. There was a significant association between previously having DM complications and adherence to diabetes medications (p = 0.000), where it was observed that most of the participants who experienced complications of DM (18.3%) had a higher rate of adherence.

**Table 5:** The association between the characteristics of the participants and the adherence of the participant to diabetes medications. *Significant at level 0.05.

Variables		Adherence	Mostly	Sometimes	Non-Adherence	P
Age (years)	18 - 30 years	15(3.5)	9(2.1)	13(3.1)	51(12)	.000*
	31 - 40 years	15(3.5)	9(2.1)	5(1.2)	24(5.6)	
	41 - 50 years	42(9.8)	5(1.2)	11(2.6)	26(6.1)	
	51 years and older	127(29.8)	14(3.3)	13(3.1)	47(11)	
Gender	Male	99(23.2)	13(3.1)3	18(4.2)	70(16.4)	.393
	Female	100(23.5)	24(5.6)	24(5.6)	78(18.3)	
Marital status	Married	3(0.7)	25(5.9)	30(7)	93(21.8)	.000*
	Single	11(2.6)	11(2.6)	11(2.6)	46(10.8)	
	Widow	14(3.3)	1(0.2)	1(0.2)	5(1.2)	
	Divorced	3(0.7)	0(0)	0(0)	4(0.9)	
Place of Residency	Al Ahsa	69(16.2)	12(2.8)	20(4.7)	62(14.6)	.293
	Ad Dammam	50(11.7)	8(1.9)	9(2.1)	37(8.7)	
	Qatif	31(7.3)	1(0.2)	3(0.7)	13(3.1)	
	Al Khobar	17(4.0)	7(1.6)	5(1.2)	17(4)	
	Al Dhahran	14(3.3)	3(0.7)	2(0.5)	8(1.9)	
	Jubail	14(3.3)	4(0.9)	2(0.5)	4(0.9)	
	Hafer al batin	4(0.9)	2(0.5)	1(0.2)	5(1.2)	
	Ras Tanura	0(0)	0(0)	0(0)	2(0.5)	
Educational level	Elementary school	14(3.3)	3(0.7)	0(0)	4(0.9)	.248
	Intermediate school	23(5.4)	1(0.2)	4(0.9)	12(2.8)	
	High school	51(12)	9(2.1)	7(1.6)	37(8.7)	
	College	101(23.7)	23(5.4)	27(6.3)	89(20.9)	
	Post-graduate	10(2.3)	1(0.2)	4(0.9)	6(1.4)	
Occupation	Student	(0.9)4	4(0.9)	9(2.1)	27(6.3)	.000*
	Employee	(10.3)44	16(3.8)	15(3.5)	52(12.2)	
	Unemployed	(12)51	10(2.3)	11(2.6)	39(9.2)	
	Retired	100(23.5)	7(1.6)	7(1.6)	30(7)	
Monthly Income	Less than 4000 SR	41(9.6)	13(3.1)	10(2.3)	44(10.3)	.162
	4000 - 8000 SR	42(9.9)	10(2.3)	11(2.6)	36(8.5)	
	More than 8000 SR	116(27.2)	14(3.3)	21(4.9)	68(16)	
Medical Insurance	No insurance	117(27.5)	21(4.9)	19(4.5)	65(15.3)	.044
	Partial insurance	23(5.4)	6(1.4)	8(1.9)	36(8.5)	
	Full insurance	59(13.8)	10(2.3)	15(3.5)	47(11)	
Healthcare type	Public health care	149(35)	29(6.8)	27(6.3)	116(27.2)	.295
	Private healthcare	50(11.7)	8(1.9)	15(3.5)	32(7.5)	
Chronic diseases	Yes	118(27.7)	11(2.6)	13(3.1)	48(11.3)	.000*
	No	81(19)	26(6.1)	29(6.8)	100(23.5)	
Diagnosis period	less than one year	45(10.6)	16(3.8)	16(3.8)	85(20)	.000*
	From 1 to 5 years	39(9.2)	7(1.6)	16(3.8)	42(9.9)	
	From 6 to 10 years	37(8.7)	3(0.7)	5(1.2)	5(1.2)	
	More than 10 years	78(18.3)	11(2.6)	5(1.2)	16(3.8)	
Body mass index	Underweight	10(2.3)	5(1.2)	5(1.2)	13(3.1)	.478
	Normal weight	25(5.9)	4(0.9)	6(1.4)	27(6.3)	
	Overweight	32(7.5)	7(1.6)	8(1.9)	30(7)	
	Obese	132(31)	21(4.9)	23(5.4)	78(18.3)	
Hemoglobin A1c level in the last time measured	Less than 7	44(10.3)	2(0.5)	7(1.6)	22(5.2)	.004
	7 and higher	79(18.5)	10(2.3)	17(4)	43(10.1)	
	Not sure	76(17.8)	25(5.9)	18(4.2)	83(19.5)	
Following a healthy diet	Yes	76(17.8)	7(1.6)	16(3.8)	49(11.5)	.138
	No	123(28.9)	30(7)	26(6.1)	99(23.2)	
Frequency of exercise	Daily	20(4.7)	4(0.9)	7(1.6)	18(4.2)	.446
	3 - 4 days per week	61(14.3)	8(1.9)	14(3.3)	54(12.7)	
	No exercise	118(27.7)	25(5.9)	21(4.9)	76(17.8)	
Previously complaining of diabetes complications	Yes	78(18.3)	10(2.3)	12(2.8)	16(3.8)	.000*
	No	121(28.4)	27(6.3)	30(7)	132(31)	

## Discussion

The control of DM is dependent on the patient’s compliance with DM medications [18]. In this study, we wanted to find the factors that may contribute to noncompliance with DM medications in patients with T2DM. Regarding adherence in our study, less than half of the participants (46.7%) were adherent to their medications, 34.7% were not adherent to their medication, 9.9% were sometimes adherent, and 8.7% were mostly adherent to their medication. This result is compatible with several studies conducted in multiple sites in Saudi Arabia, which showed that more than half of the participants were poor or not compliant at all [10-14]. This confirms that levels of adherence to DM medications are generally unsatisfactory.

We asked participants for their opinions on several factors that we believe may contribute to noncompliance with diabetes medications and lifestyle modifications in patients with T2DM. From the list of possible factors that we provided in the questionnaire, the most chosen factor was forgetfulness (44.13%). This finding is in agreement with a study by Bisha, which reported that 54% of the participants self-reported that they forgot to take their diabetic medicine [12]. Any patient can solve this problem by putting alarms and reminders on the phone that will help them remember medication time. Physicians and dietitians must suggest this step to patients during DM education sessions. Also, it would be more controllable if family members got involved with the care plan so they could assist the patient if they forgot to take their medications. Lack of knowledge about diabetes and the importance of controlling it were the second most chosen factor, which was reported by 40.61% of the participants. Most studies have found a poor level of knowledge of the disease among DM patients in Saudi Arabia [19]. We can increase the knowledge about DM in many ways, for example, by educating people on social media, motivating them to join diabetes-supportive groups, or focusing on DM education campaigns. Side effects of the medications are the third most reported reason, selected by 39.67% of the participants. We must educate the patients that most of the drug’s side effects resolve with time. The least commonly reported factors were difficulty in getting the medication and the unavailability of the medication (7.98%) and prescriptions being not clear enough (8.45%). This can be overcome if we consider Wasfaty (Wasfaty is an advanced electronic service that aims to raise the level of health services and ensure the availability of medicines). 

There was a significant association between age and adherence to diabetes medications, where it was observed that the older the participants, the higher the rate of adherence. Some studies agreed with our results [10,14]. On the contrary, some studies showed no significance [12,13]. We think that older people tend to have more wisdom and experience, so they are more aware of diabetes seriousness.

There was no significant association between gender and adherence to diabetes medications in our study. Some studies also showed no significance [12,13]. On the other hand, some studies reported that females were more adherent [11,20], while some studies pointed out males [21]. This difference may be due to geographical variations in the education and social factors between sites.

In our study, there was a significant association between marital status and adherence to diabetes medications, where it was observed that widow participants (3.3%) followed by single participants (2.6%) had a higher rate of adherence. This was unexpected because studies reported that married people adhered significantly more than singles due to spouse support [14,22,23]. However, a few studies showed no significance at all [11,12].

There was no significant association between place of residency and adherence to diabetes medication in this study. In contrast, there was a significant rural-urban difference in the noncompliance rate among diabetic patients in studies done in Alhasa [11] and Jazan [13] in 2012 and 2017, respectively. Noncompliance in the urban population was significantly higher than in the rural population. This difference may be due to various lifestyles. Urban residents tend to be more sedentary with relatively poor dietary habits compared to the rural population [13].

In this study, there was no significant association between educational level and adherence to diabetes medication. These findings are similar to results from other reviews conducted in Jazan [13] and Alkhobar [10] in 2017 and 2019, respectively. However, these results are inconsistent with the results from other reviews. A study conducted in Alhasa in 2012 showed that higher educational levels of patients were found to be significantly associated with a higher compliance rate [11]. Another study conducted in Saudi Arabia in 2017 found that college graduate patients had significantly low adherence levels compared to primary education [14]. This implies that other factors such as social, cultural, and personal traits may influence a patient’s medication-taking habit.

There was a significant association between occupation and adherence to diabetes medications, where it was observed that retired participants, followed by unemployed participants, had a higher rate of adherence. In contrast, a study conducted in 2018 about adherence to diabetes medication among diabetic patients in the Bisha governorate of Saudi Arabia showed that employment was predicted to be an important factor related to high adherence [12]. In the Jazan study, there was no statistically significant relationship found between occupation status and adherence to DM treatments [13]. To avoid controversy, further prospective studies are recommended to determine the possible contribution of occupational status to patients’ adherence.

There was no significant association between monthly income and adherence to diabetes medications. The same findings have been documented in two other studies [10,12]. We assume these results are because all people in Saudi Arabia have free access to all levels of public healthcare services.

There was no significant association between medical insurance and adherence to diabetes medication in our study. In contrast, another study conducted in Southwest Nigeria showed that National Health Insurance might influence medication adherence. The insured in the Southwest Nigeria study had significantly higher medication adherence than their uninsured counterparts [24]. We think that our result is primarily due to the quality of healthcare provided in Saudi Arabia, regardless of insurance coverage.

In our study, there was no significant association between healthcare type and adherence to diabetes medications. The same findings were established in another study conducted in Asia, which showed that there were no significant differences in medication adherence between private and public healthcare [25]. On the contrary, a previous study with similar objectives conducted in an urban area of Sri Lanka showed 35.8% adherence to public healthcare compared to 12.6% to private healthcare [25]. Currently, Saudi citizens and expatriates working in the public sector receive free healthcare through the Ministry of Health. This confirms our theory about healthcare quality provided by the Ministry of Health in Saudi Arabia.

There was a significant association between having chronic diseases and adherence to diabetes medications, where it was observed that participants with comorbidities had a higher rate of adherence than those with no comorbidities. This finding is consistent with previous literature conducted in Switzerland, where patients with comorbidities had a 43% increased level of compliance with their medications compared to those with no comorbidity [26]. A possible explanation for this result is that patients with comorbid diseases on multiple prescribed medications may have been instructed clearly on the right way of using medicines by their healthcare providers [10]. On the other hand, this outcome is contrary to previous studies from Bisha and Malaysia, which reported that compliance decreased significantly in patients with comorbidities [12,27].

The diagnosis period correlated positively with medication adherence as the ﬁndings suggest that patients tended to be more compliant as time passed since the diagnosis of diabetes. A similar study done in Southern India found that with every single passing year since the diagnosis of diabetes, patients had a 2.1% higher likelihood of compliance [28]. It can thus be assumed that the disease period plays a role in patients’ awareness and, hence, their adherence behavior.

In this study, there was no association between noncompliance and BMI. This finding is consistent with a previous study conducted in Riyadh, which revealed that BMI was not a significant predictor of diabetes medication compliance [29]. A possible explanation for this may be due to different behaviors and lifestyles, as well as the physical activities of the participants.

There was a significant association between previous complaints of DM complications and adherence to diabetes medications, where it was observed that most participants who experienced complications of DM had a higher rate of adherence. This seems logical. Patients who experienced the worst of diabetes will tend to be more responsible and careful about their health and DM control.

Dietary change is necessary for people with T2DM [30]. Unfortunately, most of the patients in this study were overweight or obese, which was found to be related to their adherence to dietary recommendations and exercise routines. Regarding following a healthy diet, 65.3% of the participants reported that they do not follow a healthy diet, while only 34.7% reported they do. These results are fairly better than the results of similar research [8]. Only 21.0% of patients adhered to the recommended diet [8]. Regarding the frequency of exercise, 56.4% were not exercising while 32.2% exercised three to four days per week, and only 11.5% exercised every day. These results are slightly better than the results of similar research [8]. Only 15.2% of participants reported adhering well to the suggested levels of physical activity, and 21.0% reported adhering partially, while the majority (63.8%) reported not adhering to any exercise recommendations at all [8]. Despite that, it is still an unsatisfactory result. It is unquestionably necessary to increase patient compliance with lifestyle modifications through the hospital- and community-based awareness programs that emphasize the significance of lifestyle alteration for diabetes patients and their families to receive ongoing support and consistency with their diabetes treatment.

Limitations of the study

One of the main limitations was the study approval, which restricted the sample collection tool to an online questionnaire. There were few studies matching ours in Saudi Arabia. Therefore, we were not able to bring more studies to discuss and compare with ours. Bias in participant selection could occur since most of the participants have a college degree and are, in general, more familiar with online surveys than lower educational levels.

## Conclusions

This study showed that the level of adherence to DM medications among T2DM patients in the eastern province was suboptimal. Although free medicines were available with a high level of healthcare access through government PHCCs, poor adherence was observed.

The results of this study showed the factors that affect T2DM and contribute to noncompliance with lifestyle modification and medications in the eastern province of Saudi Arabia from the participants’ perspective. The most reported factors were forgetfulness, lack of knowledge about diabetes and the importance of controlling it, side effects of the medications, and difficulty in following a healthy diet. The least commonly reported factors were difficulty in getting the medication and the unavailability of the medication. This study highlighted that medication adherence might be affected by age, marital status, occupation, chronic diseases, diagnosis period, and previous complaints of DM complications. Gender, place of residency, educational level, monthly income, medical insurance, healthcare type, BMI, HbA1c, following a healthy diet, and frequency of exercise were not significantly associated with adherence to diabetes medications.

Our recommendations are to increase the education level about the disease, the importance of adherence to diabetes medications, and the different ways to limit forgetfulness through intensive awareness campaigns. A possible idea is to try to include this in school curriculums to create a more diabetes-aware generation.

Regarding lifestyle modification, this study showed that the level of adherence to a healthy diet and exercise among T2DM patients in the eastern province was low and unsatisfactory. We recommend increasing the awareness of lifestyle modifications such as exercise and following a healthy diet with regular follow-ups in the dietitian clinic.

Our recommendation is to measure the presence of dietician clinics, patient relationships with their healthcare providers, and their effect on compliance with DM medications. Further research is needed to include other factors that could influence adherence, such as patient-healthcare provider communication. Moreover, it is suggested that PHCCs discuss with noncompliant patients the reasons that prevent them from adhering to their medication and lifestyle modifications as part of their care plan.

This study showed that the level of adherence to DM medications among T2DM patients in the eastern province is suboptimal. Even when free medicines were available with a high level of healthcare access through government PHCCs, our study demonstrated poor adherence.

The result of this study showed the most contributing factors that affect T2DM and contribute to non-compliance to lifestyle modification and medications in the Eastern province of Saudi Arabia in the opinion of the participants. The most reported factors were forgetfulness, lack of knowledge about diabetes and the importance of controlling it, side effects of the medications, and the hardness of following a healthy diet. The least commonly featured parameters roam around the difficulty of attainment of the medication process as well as its unavailability. This study highlights that medication adherence may be affected by age, marital status, occupation, chronic diseases, diagnosis period and previously having DM complications. There was no significant association between gender, place of residency, educational level, monthly income, medical insurance, health care type, BMI, HbA1C, following a healthy diet, and frequency of exercise with adherence to diabetes medications.

Our recommendations are to increase the education level about the disease, the importance of adherence to diabetes medications, and the different ways to limit forgetfulness. Through intensive awareness campaigns. A possible idea is trying to include this in school curriculums to create a more diabetes-aware generation.

Regarding lifestyle modification, this study showed that the level of adherence to a healthy diet and exercise among T2DM patients in the eastern province is low and unsatisfactory. We recommend increasing the awareness of lifestyle modifications such as exercise and following a healthy diet with a regular follow-up in the dietitian clinic.

Our recommendation is to measure the presence of dietician clinics, patient relationships with their HCPs, and their effect on compliance with DM medications. Also, further research is recommended to include other factors that could influence adherence, such as patient-healthcare provider communication. Moreover, it is recommended that PHCCs Discuss with noncompliant patients the reasons that prevent them from adhering to their medication and lifestyle modifications as part of their care plan.
